# Experimental and Numerical Investigation on Dragonfly Wing and Body Motion during Voluntary Take-off

**DOI:** 10.1038/s41598-018-19237-w

**Published:** 2018-01-17

**Authors:** Qiushi Li, Mengzong Zheng, Tianyu Pan, Guanting Su

**Affiliations:** 1 National Key Laboratory of Science and Technology on Aero-Engine Aero-thermodynamics Collaborative Innovation Center of Advanced Aero-Engine, School of Energy and Power Engineering, Beihang University, Beijing, China; 2 Department of Mechanical Engineering and Materials Science, Duke University, Durham, NC 27708 USA

## Abstract

We present a detailed analysis of the voluntary take-off procedure of a dragonfly. The motions of the body and wings are recorded using two high-speed cameras at Beihang University. The experimental results show that the dragonfly becomes airborne after approximately one wingbeat and then leaves the ground. During this process, the maximum vertical acceleration could reach 20 m/s^2^. Evidence also shows that acceleration is generated only by the aerodynamic force induced by the flapping of wings. The dragonfly voluntary take-off procedure is divided into four phases with distinctive features. The variation in phase difference between the forewing and hindwing and angle of attack in the down-stroke are calculated to explain the different features of the four phases. In terms of the key parameters of flapping, the phase difference increases from approximately 0 to 110 degrees; the angle of attack in down-stroke reaches the maximum at first and then decreases in the following take-off procedure. Due to experimental limitations, 2-D simulations are conducted using the immersed boundary method. The results indicate that the phase difference and the angle of attack are highly correlated with the unsteady fluid field around the dragonfly’s wings and body, which determines the generation of aerodynamic forces.

## Introduction

Current aircrafts and remote air vehicles are too large to operate in some special civil applications, such as search and rescue operations inside buildings, but a micro air vehicle (MAV), with a size smaller than 150 mm and a weight less than 50 g^1,2^, can be used.

Inspired by the motion of tiny flying insects, flapping has been implemented as the primary mechanism of force generation in almost all developed MAVs to satisfy the abovementioned requirements^3,4^. Studies on the motion of the body and wings of insects in different flight states have shown that flapping could be the most efficient and most powerful flying mode due to the notable viscous effect under low Reynolds number situations^5–7^. However, the mechanisms of the generation of great aerodynamic forces by flapping insects in nature are still unknown.

During the take-off procedure, great force is generated so that the insect can overcome gravity and quickly leave the ground. In previous studies, based on photos and short videos taken during the take-off process, several modes of body motion and flapping were obtained:

*Flapping with leg jump*. Pond^8^ studied the take-off of a species of locust, *Schistocerca gregaria*, and found that the locusts first jump into the air before flapping their wings. For milkweed bugs, *Oncopeltus*, Govind and Dandy^9^ showed that the take-off jump (executed by a complex of trochanteral depressor muscles in the legs) occurred after two wingbeats. For the fruit fly, *Drosophila melanogaster*^10–12^, two types of flight initiation were captured. Under strong visual stimuli, the take-off process is similar to that of the locust; otherwise, the take-off process is the same as that of the milkweed bug.

*Clap and fling*. Sunada^13^ measured the kinematics of the wings and body during the take-off of a butterfly using a high-speed video camera and then developed a vortex method to compute the aerodynamic force on the flapping wings. They found that the first peak of lift-off is generated by the opening motion of the two wings.

*Slow take-off*. Chen *et al*.^14^ examined the voluntary take-offs of droneflies, *Eristalis tenax*, and showed that droneflies do not jump but only flap the wings to lift themselves into the air. As a result, the take-off process takes a longer time.

In sum, the combined use of flapping and leg jumps results in a fast take-off process. However, it is difficult to design and manufacture a system using this mechanism for MAVs at the current stage.

Among the many insects with outstanding flying abilities, dragonflies possess unique flight characteristics and strong maneuvering abilities^15^. Experimental studies on the gliding, hovering and uniform forward flight of dragonflies have been reported^16–18^. In gliding flight, the wings of dragonflies remain almost immobile, and the forewing and the hindwing are almost on the same plane. In hovering and uniform forward flight, the dragonfly flaps the wings asynchronously with a phase difference between the forewing and hindwing of approximately 90° to 150°, and the positional angle and angle of attack of the wing are hardly different between each process. Sun and Lan^19^ calculated the lift and thrust coefficient of dragonflies with different phase differences between the forewing and hindwing using a three-dimensional numerical simulation method. They showed that synchronized flapping can generate the largest lift and that the 90° phase difference between the forewing and hindwing is helpful for thrust. However, the take-off behavior of dragonflies has not been well studied, which is likely the most important and most powerful process. In this paper, the process of voluntary take-off of a dragonfly (*Pantala flavescens*) is recorded using two high-speed cameras and a three-dimensional reconstruction technique to study the real motion of the body and wings. Based on these results, the kinematics of the body and wings are calculated to illustrate the link between wing flapping and body movement, which results in an understanding of the voluntary take-off procedure of dragonflies.

Based on the take-off mechanism, the wingbeat of dragonflies could inform a mode of rapid take-off solely relying on flapping for MAVs. To further study why the wingbeat of a dragonfly can induce such high aerodynamic lift and to determine the key parameters related to the generation of aerodynamic lift, two-dimensional numerical simulation based on the IBM (immersed boundary method) is carried out for various flapping phase differences (between the forewing and hindwing) and angles of attack in the down-stroke.

## Experimental Results and Discussions

Thirty dragonflies were tested. The maximum and minimum masses were 359 mg and 331 mg, respectively, with a relative difference of approximately 8%. The maximum and minimum body lengths were 49.4 mm and 47.9 mm, respectively, with a relative difference of approximately 3%. Due to these small differences in mass and size, the effects of mass and size were not taken into account.

During the experiment, parameters related to the voluntary take-off procedure were only recorded for eight individuals. Some similar phenomena were observed: First, dragonflies tended to leave the ground after one wingbeat. Second, dragonflies started to flap their wings synchronously, and the phase difference increased from 0 degrees during the subsequent wingbeats. Of the eight individuals, only three could be further analyzed because all the recorded parameters could be recognized only for these three individuals. In some cases, the wings were hidden behind other wings or the dragonfly flew outside the focused plane of the cameras.

For these three cases (labelled as D1, D2 and D3), all the analyzed results are presented, which is shown on Table 1.

**Table 1:** Some experimental observation parameters of the dragonflies. D1, D2 and D3, dragonflies 1, 2 and 3, respectively; FW, forewing; HW, hindwing.

Individual	Time of Take-off (ms)	Displacement (m)		Average Frequency (Hz)		Average Stroke Amplitude (degrees)		the Phase Difference (degrees)		Maximum Value of the Angle of Attack in Down-stroke (degrees)	
		Upward	Forward	FW	HW	FW	HW	Min	Max	FW	HW
D1	350	0.25	0.12	25.8	27.3	73.3	65.9	0	109.5	86	84
D2	380	0.31	0.13	25.4	27.1	74.5	65.4	0	109.2	86	88
D3	300	0.23	0.11	26.1	27.4	74.2	66.1	0	110.6	87	86

The kinematic parameters share very similar values: In terms of the time of take-off procedure, for D1, D2 and D3, it is 350 ms, 380 ms and 300 ms, respectively. In terms of displacement, the largest differences are 0.08 m for upward movement and 0.02 m for forward movement. For average frequency, the largest differences are 0.7 Hz for forewing and 0.3 Hz for hindwing. For average stroke amplitude, the largest difference are 1.2 degree for forewing and 0.7 degree for hindwing. For the range of the phase difference, the minimum values are all 0 while the maximum fluctuating in the range from 109.2 degree to 110.6 degree. In the end, for the maximum value of the angle of attack in down-stroke, the largest differences are 1 degree for forewing and 4 degree for hindwing. Therefore, only the experimental results from one individual (D1) will be further analyzed to determine the mechanisms of dragonfly voluntary take-off.

### Voluntary take-off process

**Figure 1:**
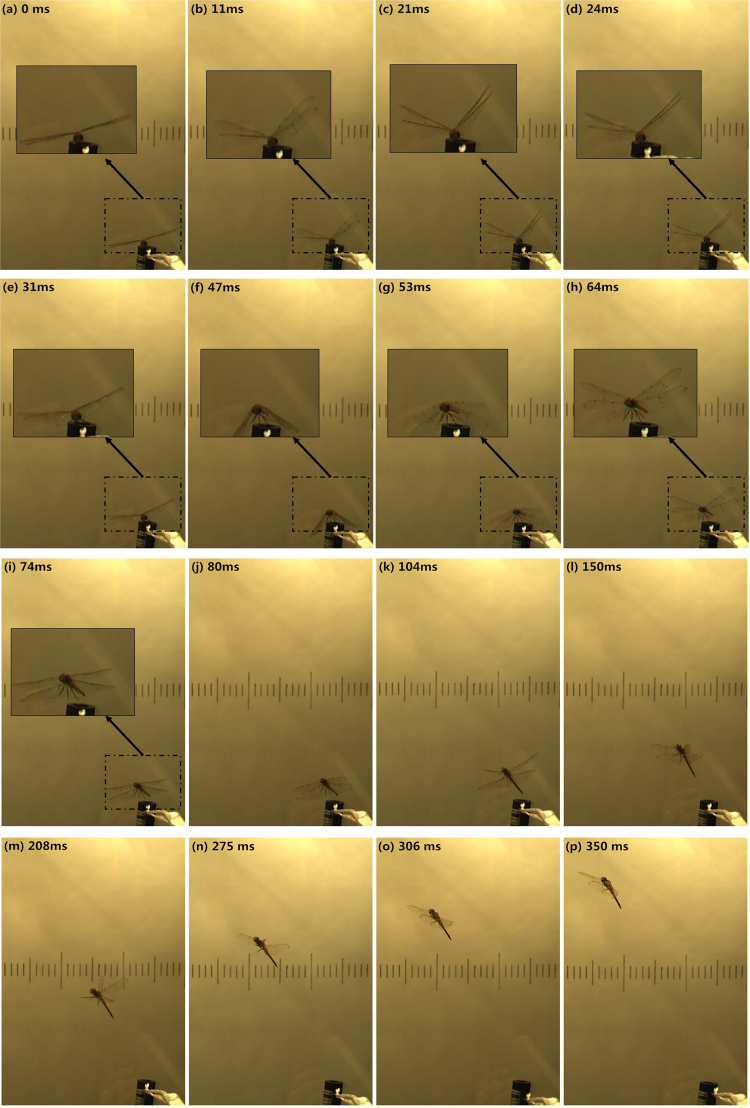
Video sequences (16 typical moments) of a dragonfly (D1) take-off. Two high-speed cameras are used in the experiment (the experimental scheme is detailed in the Methodology Section), and the view sequences above are from high-speed camera 2. The unit of time is ms. The vertical lines in the background are a reference scale for three-dimensional reconstruction.

There are approximately 8 stroke cycles in the voluntary take-off process of D1, which lasts for approximately 0.4 s. The sampling frequency of the camera is 1000 Hz, so 400 frames are recorded. After individual analysis of these 400 frames, 16 typical moments are chosen and displayed to describe the voluntary take-off procedure of the dragonfly (Fig. 1).

In Fig. 1, the 16 typical moments are labeled **a** to **p**. The time of moment **a** (corresponding to the beginning of wing flapping) is set to be 0.

For the entire process, four phases with distinctive features can be identified:

**Phase I**: from 0 to 47 ms. At 0 ms (moment **a**) the dragonfly starts to raise the fore and hind wings synchronously. Later, at 21 ms (moment **c**), both the forewing and hindwing reach the top position for the first time and start to flap down synchronously, while the body starts to rise up. Moment **b** (11 ms) is one point in the first up-stroke, and moment **e** (31 ms) is one point in the first down-stroke.

**Phase II**: from 47 ms to 80 ms. At 47 ms (moment **f**) the first down-stroke is almost over, and the dragonfly leaves the ground. From 47 to 80 ms, the dragonfly flaps both the fore wing and hind wing synchronously for another stroke (verified by moments **f** to **j**). Moment **g** (53 ms) is one point in the second up-stroke. At 64 ms (moment **h**), both the fore wing and hind wing reach the top position for the second time.

**Figure 2:**
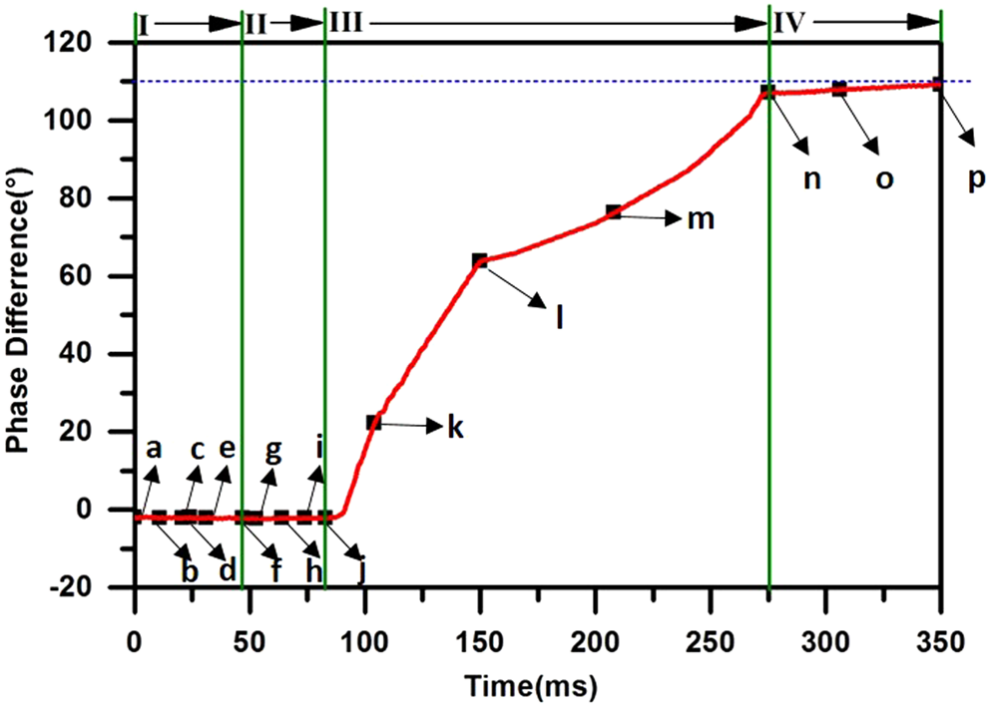
The variation in the phase difference during the entire process of voluntary take-off.

**Phase III**: from 80 to 275 ms. At 80 ms, the flapping motion of the hind wing starts to become faster than that of the fore wing. As a result, the phase difference increases between the motion of the fore wing and hind wing. During the entire phase, the phase difference increases gradually from approximately 0 degrees to 110 degrees (the variation in phase difference is plotted in Fig. 2). Meanwhile, the rate of variation in the phase difference also changes; from 80 ms to 150 ms (moments **j** to **l**), the phase difference increases much more quickly than from 150 ms to 275 ms (moments **l** to **n**).

**Phase IV**: from 275 to 350 ms. In this phase (moments **n** to **p**), the phase difference is fixed at approximately 110 degrees. Finally, the take-off process is over, and the dragonfly starts to fly forward.

**Figure 3:**
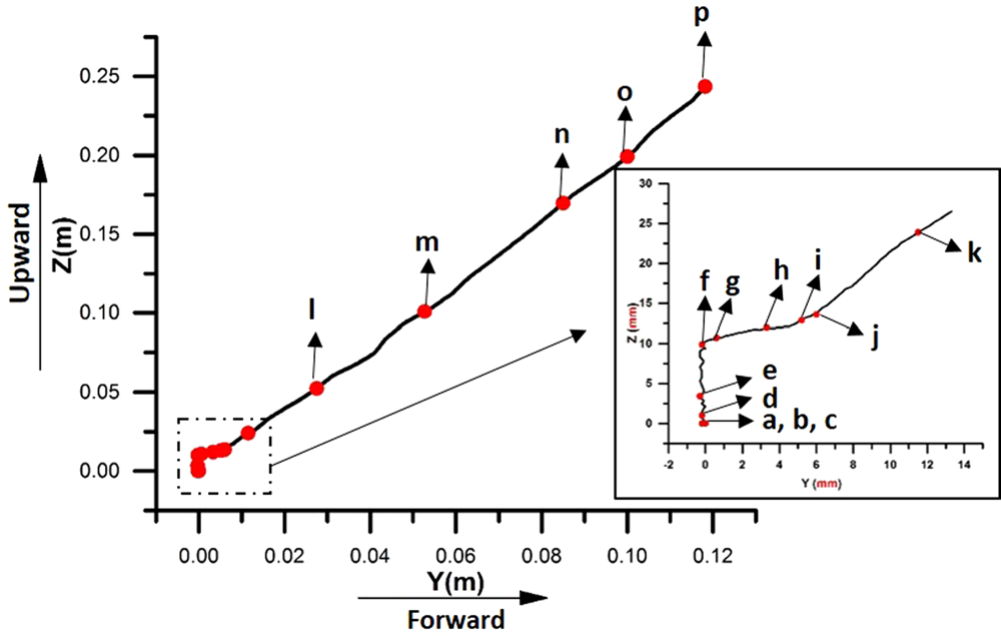
Displacement of the dragonfly centroid in the upward direction (along the Z-axis) and the forward direction (along the Y-axis).

After three-dimensional reconstruction based on all recorded frames, the displacement of the body centroid in a relative coordinate system, which is detailed in the Methodology Section, is obtained and plotted in Fig. 3. Note that the displacement along the X-axis is very close to zero throughout the entire process of take-off, so only the displacements along the Y-axis and Z-axis are plotted. The coordinates at 0 ms are set to (0, 0, 0). All the typical moments consistent with Fig. 2 are also labeled.

In **Phase I** (moments **a** to **f**), only displacement in the upward direction (along the Z-axis) can be detected. In the following **Phases II** to **IV** (moments **f** to **p**), movements in both the upward direction (along the Z-axis) and the forward direction (along the Y-axis) are captured. The displacements in both directions increase at a nearly constant rate. As a result, from 0 to 350 ms, the dragonfly flies approximately 0.25 m upward and moves approximately 0.12 m forward during the entire process of voluntary take-off.

## Body kinematics

**Figure 4:**
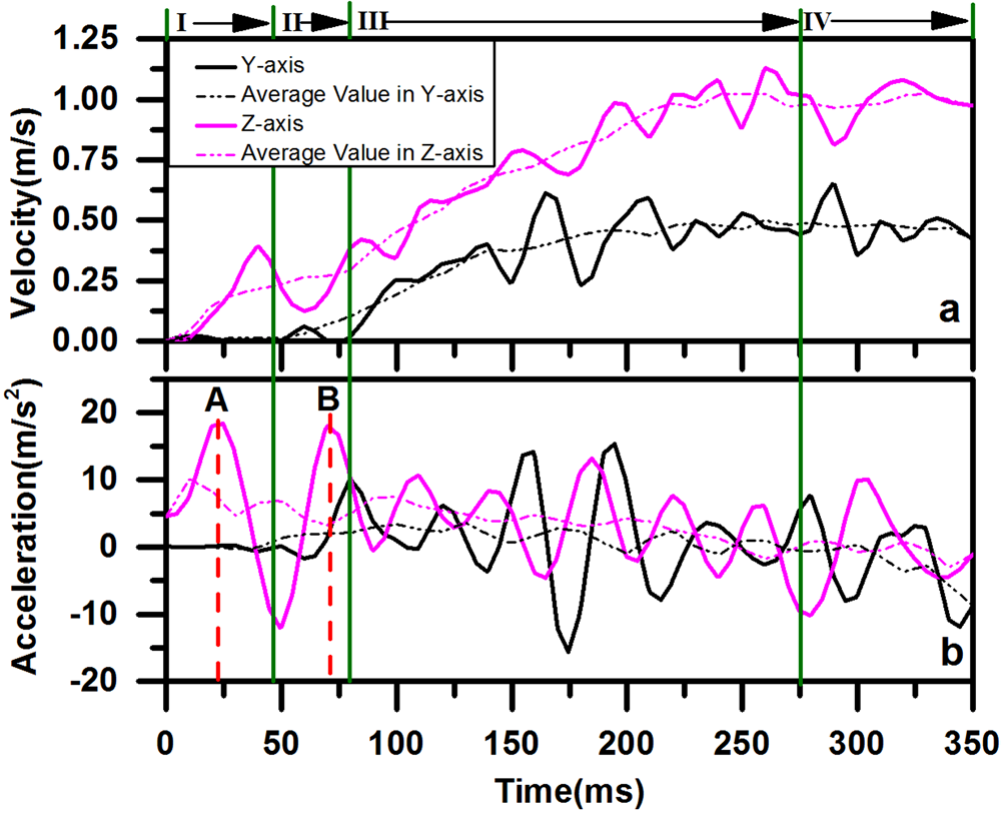
Time histories of body kinematics of D1. (**a**) Velocity of the dragonfly centroid. (**b**) Acceleration of the dragonfly centroid.

To further study the voluntary take-off process, the body kinematics were calculated according to the displacements. The first derivative of the displacement-time function yields the velocity of the centroid, and the second derivative yields the acceleration of the centroid. The first and second derivatives of the displacements are calculated in both the Y-axis and Z-axis directions. Then, the derived velocity and acceleration are low-pass filtered to remove noise, as shown in Fig. 4. Two salient findings are noted for the body kinematics of a dragonfly:

First, the variations in both velocity and acceleration correspond to different features of motion in the four phases. In **Phase I**, the dragonfly stays on the ground, so there is neither displacement nor acceleration along the Y-axis. In **Phase II**, both velocity and acceleration along the Y-axis begin to increase when the dragonfly starts to take off. However, the velocity and acceleration remain small in this phase. In **Phase III**, the velocities along the Z-axis and Y-axis keep increasing, but the accelerations along both directions finally decrease. This distinguished change may be induced by the change in the phase difference between the fore wing and hind wing, which will be further discussed in the simulation section of this study. In **Phase IV**, velocities and accelerations remain constant, corresponding to the constant phase difference.

Second, among the 8 peaks of acceleration along the Z-axis detected during this process, the first and second peaks labeled by **A** and **B** in Fig. 4b have the same largest value (20 m/s^2^, twice the acceleration of gravity). The first peak **A** results from the first down-stroke corresponding to moment **d** in Fig. 1, and the second peak **B** is induced by the second down-stroke corresponding to moment **i** in Fig. 1. Note that the acceleration curve does not show a sudden rise in the first two wingbeats. Thus, it can be verified that leg leaping is absent during the voluntary take-off of the dragonfly.

### Wing kinematics

**Figure 5:**
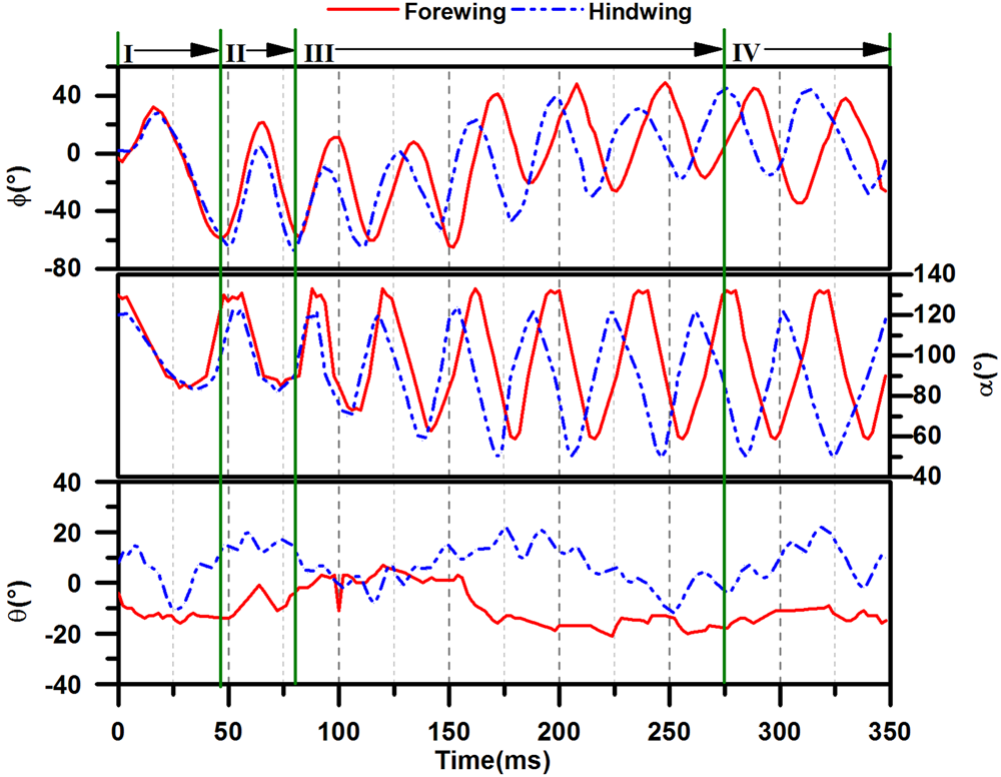
Time histories of wing flapping motion. *φ*, positional angle; *θ*, stroke deviation angle; *α*, the angle of attack of the wing, which are detailed defined in Methodology Section. t = 0 is the time when the wings of the insect start to move, corresponding to moment **a**.

Figure 5 shows the measured angles of wing flapping as functions of time for the fore wing and hind wing. During the voluntary take-off of the dragonfly, the positional angle *ϕ* fluctuates, and the variation in the phase difference can be clearly seen. Let *Φ* and $\bar{\varphi }$ denote the stroke amplitude and mean stroke angle, respectively; they are defined by “$\Phi ={\varphi }_{max}-{\varphi }_{min}$ and $\bar{\varphi }=\frac{{\varphi }_{max}+{\varphi }_{min}}{2}$, where ${\varphi }_{max}$ and ${\varphi }_{min}$ are the maximum and minimum values of *ϕ*, respectively.

Figure 5 also shows that for both wings, the angles of attack (*α*) at the bottom of the down-stroke are approximately 85 degrees in **Phase I** and **Phase II**, much greater than those in the following phases. Actually, in **Phase III**, the angle of attack decreases from 85 degrees. Finally, in **Phase IV**, the angle of attack remains approximately 60 degrees.

**Figure 6:**
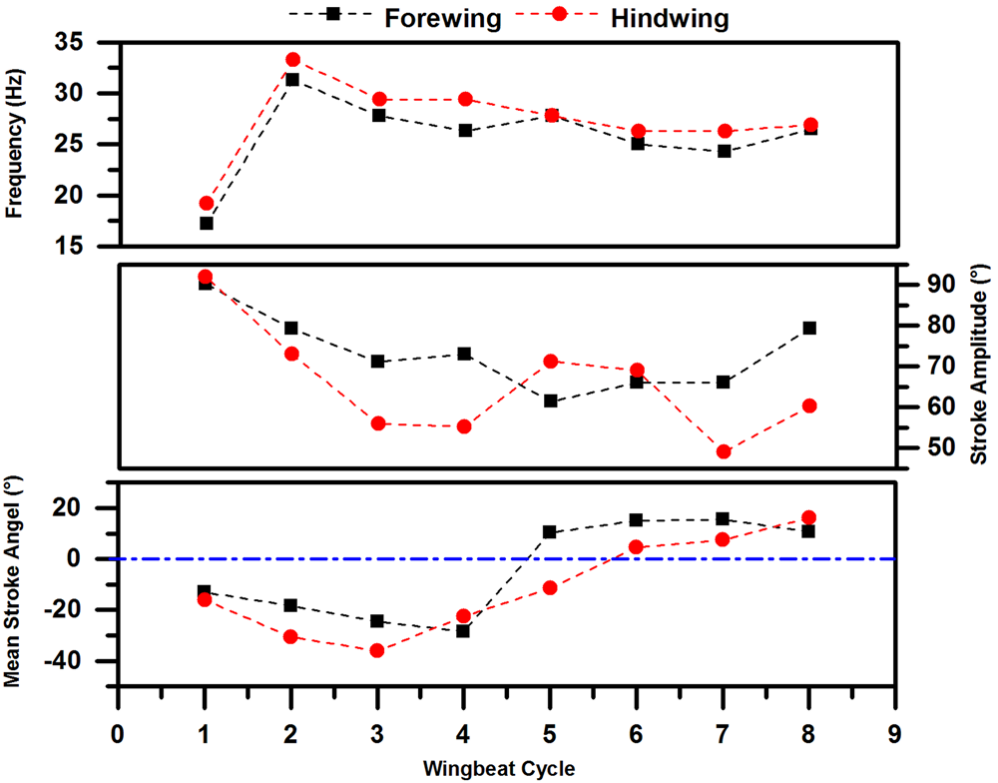
The frequency of wingbeat (***n***), the amplitude of stroke (*Φ*) and the mean stroke angle ($\bar{\varphi }$) in each wingbeat cycle during the voluntary take-off of a dragonfly.

To further analyze the wing flapping motions in Fig. 5, the frequency of wingbeat (***n***; estimated based on the time period between two adjacent peaks of the angle fluctuation), the amplitude of stroke (*Φ*) and the mean stroke angle ($\bar{\varphi }$) are calculated and summarized in Fig. 6. For the first two wingbeats, the estimated frequency is 17.2 Hz for the fore wing and 19.2 Hz for the hind wing. After that, the frequency fluctuates around 30 Hz in each wingbeat cycle.

The amplitudes of the stroke of both the fore wing and hind wing reach maximum values of 90.2 degrees and 92.0 degrees in the first wingbeat, respectively. Later, the stroke amplitude is almost constant. For the fore wing, the amplitude of stroke goes down until the 6^th^ wingbeat cycle and then increases. For the hind wing, the amplitude decreases to the minimum at the 4^th^ wingbeat cycle and then increases again. The mean stroke angles of both the fore wing and hind wing change from negative to positive values.

### Summary of this section

The voluntary take-off of the dragonfly can be divided into four phases according to the distinctive features of body motion, body kinetics and wing motion. From **Phases I** to **IV**, the dragonfly is airborne in one wingbeat and then starts to take-off. The maximum vertical acceleration is close to 20 m/s^2^. The aforementioned experimental results show that the acceleration is generated by only the aerodynamic force. The phase difference between the forewing and hindwing increases from approximately 0 degrees to 110 degrees. The angle of attack (*α*) at the bottom point of the down-stroke of both wings is significantly greater at first and then decreases. During the four phases of the take-off procedure, the body motion of the dragonfly varies tremendously, indicating a variation in the aerodynamic forces generated by the flapping wings. This variation in force is accompanied by the change in two kinematic parameters of the wingbeat, namely, the phase difference and the angle of attack, as mentioned above. It would be of interest to investigate their influence on the aerodynamic forces.

## Results of CFD (Computational Fluid Dynamics) simulations and discussions

Based on the experimental results, the kinematics of the voluntary take-off process of a dragonfly are described. In this section, two-dimensional immersed boundary computational fluid dynamics is used to investigate the key parameters of wingbeat related to the aerodynamic lift in the different phases of the take-off process.

A few studies have been done to assess the unsteady large-lift mechanisms of flapping insect wings^20–23^. When the insect wing is flapping, a leading-edge vortex (LEV) is formed, but the vortex does not detach from the wing in an entire down-stroke or up-stroke. Thus, a large aerodynamic force produced by the vortex can be maintained. Therefore, the large aerodynamic force in insect flight is closely related to such a vortex. This mechanism is also called the delayed-stall mechanism because the stall is delayed permanently. It is also known that the aerodynamic lift is produced mostly during the down-stroke, while the drag is produced mostly during the up-stroke.

In the experiments mentioned above, we found that the phase difference and angle of attack in the down-stroke vary during the voluntary take-off of the dragonfly. The influences of these two parameters on the vortex structure and the aerodynamic forces are analyzed with the two-dimensional immersed boundary method.

Some parameters required in the simulations are obtained based on the experimental results, including the dimensionless flapping amplitude “*A*_*f*_ = *A*_*h*_ = 2.5” (the difference between the forewing and hindwing is ignored), the flapping frequency “*f* = 30 Hz” (assuming that the flapping frequencies of both wings are the same) and the stroke plane angle of the fore and hind wings (*β*_*f*_ = 67.2 degrees and *β*_*h*_ = 65.4 degrees, respectively). In addition, different values of φ and α_down-stroke_ are defined according to the actual situation to analyze the interaction of the dragonfly wings.

### Effect of angle of attack on the down-stroke

Two cases are simulated with different angles of attack in the down-stroke. Case 1: *φ* = 0 degrees, *α*_*down-stroke*_ = 86 degrees. Case 2: *φ* = 0 degrees, *α*_*down-stroke*_ = 60 degrees. The other conditions are the same.

**Figure 7:**
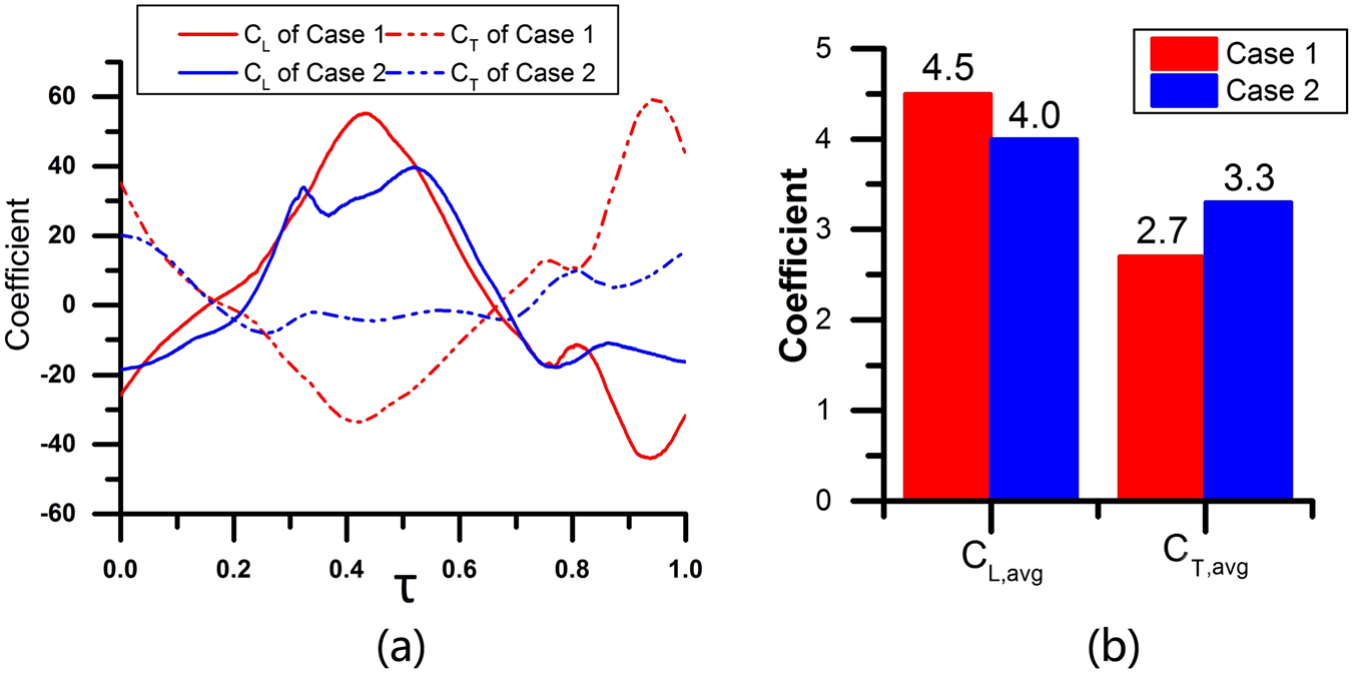
(**a**) *C*_*L*_ and *C*_*T*_ of wings during one wingbeat of Case 1 and Case 2 (the horizontal axis is dimensionless cycle time *τ*); (**b**) cycle averaged lift and thrust coefficient of Case 1 and Case 2.

The lift coefficient (*C*_*L*_) and thrust coefficient (*C*_*T*_) of wings during one wingbeat are calculated and integrated to derive the cycle averaged lift coefficient (*C*_*L, avg*_) and thrust coefficient (*C*_*T, avg*_), as shown in Fig. 7. Note that positive *C*_*L*_ represents lift and positive *C*_*T*_ represents thrust. Figure 7a shows that both lift and thrust coefficients in case 1 fluctuate more heavily than those in case 2. For example, the peak lift coefficient in case 1 reaches 58, while it is only approximately 40 in case 2. The trough lift coefficient is approximately −40 in case 1 but only −20 in case 2. The average lift coefficient in case 1 reaches 4.5, 12.5% greater than that in case 2, but the average thrust coefficient in case 1 is smaller than that in case 2, as presented in Fig. 7b.

**Figure 8:**
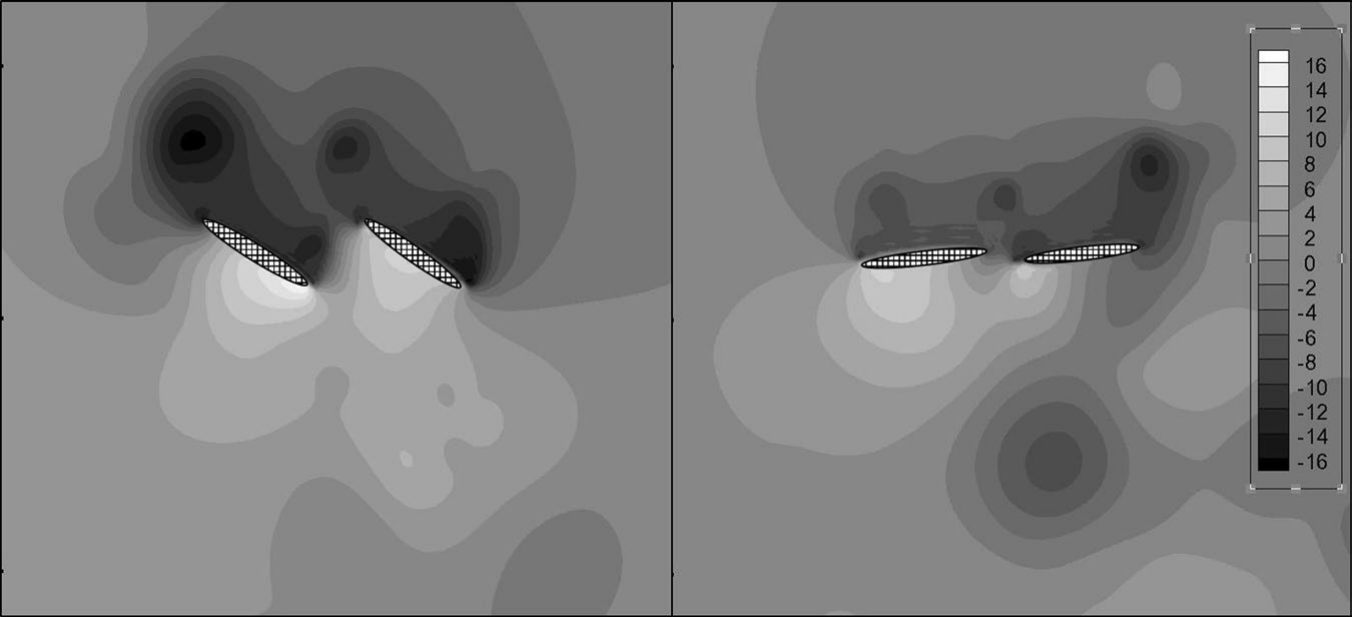
The pressure at *τ* = 0.43 of Case 1 (left) and Case 2 (right). *P* is the dimensionless pressure and *P* = *p*/*ρU*^2^, where ρ is the air density and U is the characteristic velocity. Ellipses with mesh background are the wing models. The wing model in the left side is the forewing and the other one is the hindwing in each picture.

The values of the dimensionless pressure *P* at *τ* = 0.43 in Case 1 and Case 2 are shown in Fig. 8. The pressure differences between the upward sides and downward sides of the wings result in lift. In Case 1, the scales of the high-pressure zone and low-pressure zone are much larger than those in Case 2. At the same time, the maximum normalized pressure in Case 1 is approximately 16, while it is only approximately 10 in Case 2. The minimum normalized pressure in Case 1 is approximately −17, while it is only −13 in Case 2. As a result, the lift is greater in Case 1.

For thrust, when the wing is flapping with a greater angle of attack in the down-stroke, the pressure difference contributes to the thrust, which thus largely fluctuates in Case 1, as presented in Fig. 7. A greater *α*_*down-stroke*_ leads to a greater pressure difference in the thrust-axis (horizontal direction), so the thrust of Case 1 is much smaller than that of Case 2 at *τ* = 0.43, as shown in Fig. 8. For the averaged thrust force, flapping with a lower angle of attack in the down-stroke induces larger thrust in the entire flapping cycle.

**Figure 9:**
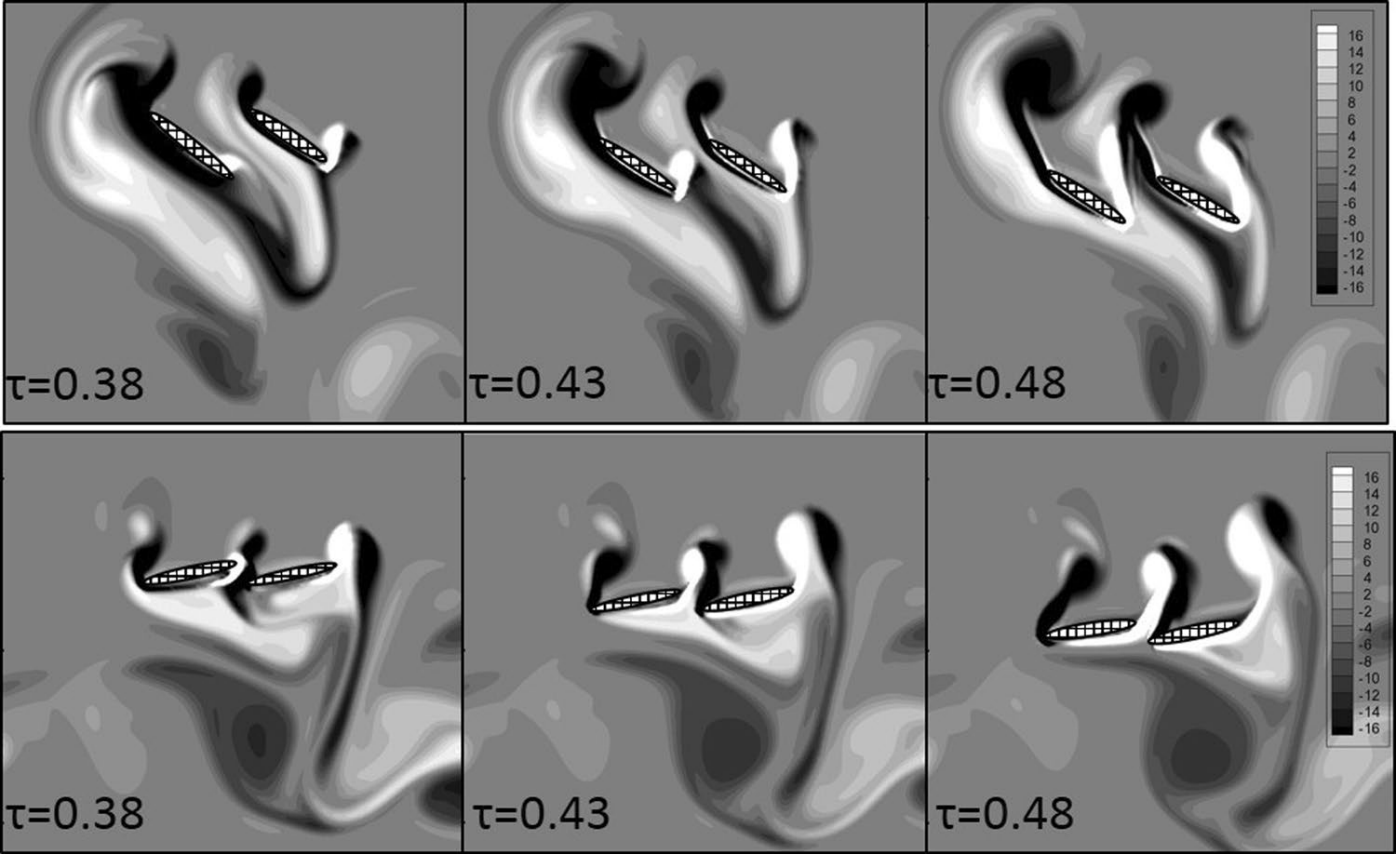
The vorticity from *τ* = 0.38 to *τ* = 0.48 of Case 1 (up) and Case 2 (down). Ellipses with mesh background are the wing models. The wing model which locates relatively at the left side is the forewing and the other one is the hindwing in each picture.

The vortex fields from *τ* = 0.38 to *τ* = 0.48 in Case 1 and Case 2 are shown in Fig. 9. In Case 1, the larger *α*_*down-stroke*_ makes it hard for the fluid to flow though the gap between the fore wing and hind wing, so the fluid on the downward side of the wings is compressed. Thus, a high-pressure zone with greater pressure and wider extent is formed on the downward sides of wings, especially near the trailing edge of the fore wing. Then, the fluid is driven forward and flows over the leading edge of the fore wing. Because a higher velocity basically suggests a lower static pressure, a low-pressure zone can be found on the upward sides of wings, especially near the leading edge of the fore wing. In Case 2, the smaller *α*_*down-stroke*_ makes it easier for the fluid to flow though the gap between the fore wing and hind wing, so the fluid on the downward sides of the wings is not blocked as much as in Case 1. Thus, the scale of the high-pressure zone on the downward sides of wings is smaller, and the pressure is lower. Meanwhile, the fluid on the downward sides overflows to the upward sides via three routes: 1) through the leading edge of the fore wing, 2) through the gap between the fore wing and hind wing, and 3) through the trailing edge of the hind wing. As a result, the velocity of the upward side is lower in Case 2, and the static pressure is higher in Case 1. The vortex field explains the greater pressure difference across the wings and why the location of the low-pressure zone and high-pressure zone are not the same in the two cases.

In sum, flapping with a greater angle of attack in the down-stroke results in greater lift, while flapping with a lower angle of attack in the down-stroke induces greater thrust but smaller lift. These results are consistent with the experimental results. At the beginning of take-off, the dragonfly requires larger lift, so it will flap the wings with a greater angle of attack in the down-stroke and then switch to a lower angle of attack in the down-stroke mode when it has already left the ground and needs to fly upward and forward.

### Effect of phase differences between the forewing and hindwing

Two other cases are also simulated with various phase differences: Case 2 (*φ* = 0 degrees, *α*_*down-stroke*_ = 60 degrees) and Case 3 (*φ* = 110 degrees, *α*_*down-stroke*_ = 60 degrees). The other conditions are the same.

**Figure 10:**
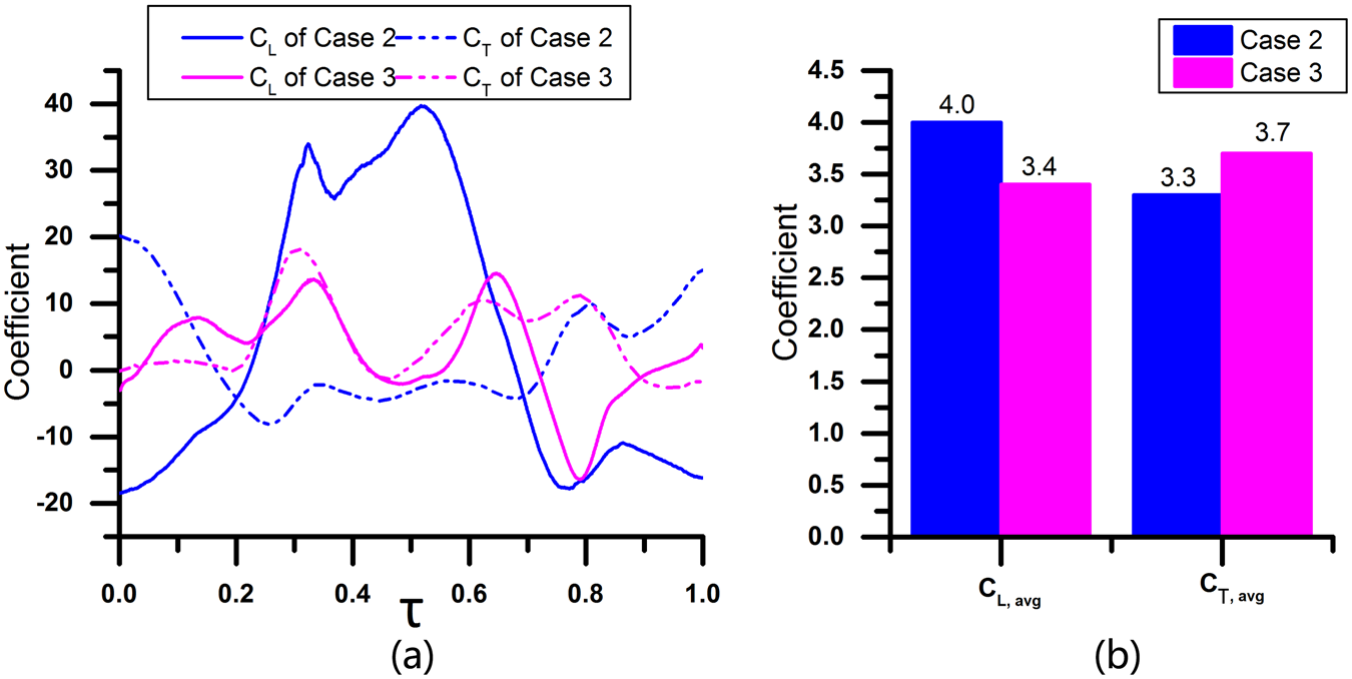
(**a**) *C*_*L*_ and *C*_*T*_ of wings during one wingbeat in Case 2 and Case 3 (the horizontal axis is dimensionless cycle time *τ*); (**b**) cycle averaged lift and thrust coefficient in Case 2 and Case 3.

The lift coefficient (*C*_*L*_) and drag coefficient (*C*_*T*_) of wings during one wingbeat, as well as the averaged lift coefficient (*C*_*L, avg*_) and averaged thrust coefficient (*C*_*T, avg*_), are shown in Fig. 10. We can speculate that the maximum *C*_*L*_ generated by synchronous flapping (Case 3) is larger than that generated by asynchronous flapping (Case 4), and the *C*_*L, avg*_ of synchronous flapping is 20.6% larger than that of asynchronous flapping.

**Figure 11:**
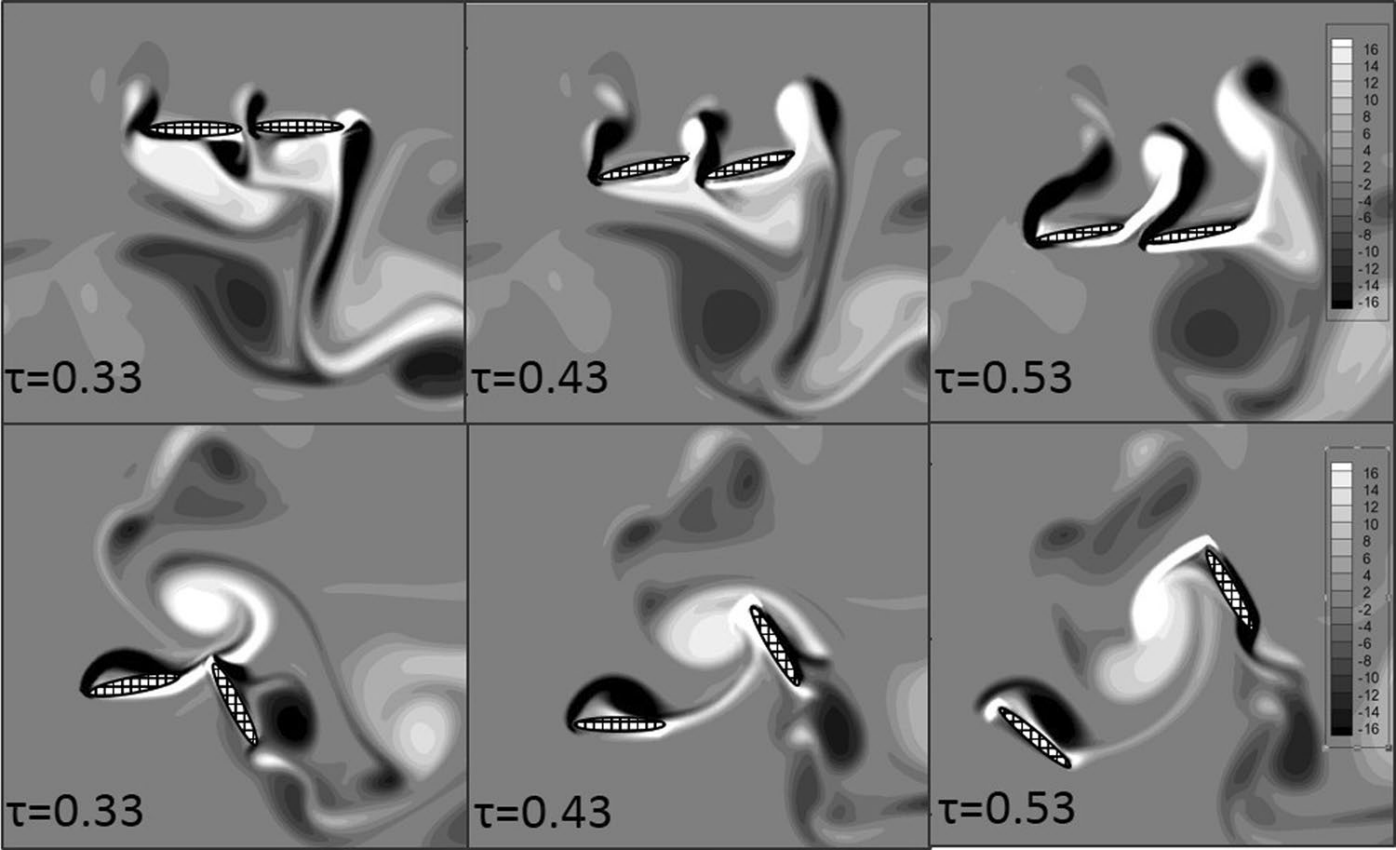
The vorticity from *τ* = 0.33 to *τ* = 0.53 of Case 2 (up) and Case 3 (down). Ellipses with mesh background are the wing models. The wing model in the left side is the forewing and the other one is the hindwing in each picture.

In Fig. 10(a), the *C*_*L*_ and *C*_*T*_ of Case 3 display smaller fluctuations than those of Case 2. The vortex fields in Case 2 and Case 3 from *τ* = 0.33 to *τ* = 0.53 are also shown in Fig. 11. In Case 3, both the fore wing and hind wing are flapping in the same direction; as a result, the directions of the aerodynamic forces generated by both the fore wing and hind wing are the same. In Case 4, asynchronous flapping of the fore wing and hind wing leads to opposite directions of the aerodynamic forces generated by the fore wing and hind wing; as a result, the averaged aerodynamic force of both the fore wing and hind wing is more stable.

The thrust generated by synchronous flapping fluctuates with greater amplitude; it is positive when the wings are flapping up and negative when the wings are flapping down. However, in Case 4, the thrust induced by asynchronous flapping almost remains entirely positive. Thus, the averaged thrust is greater in Case 4 than in Case 3.

In sum, the lift generated by synchronous flapping is greater, and when the phase difference between the fore wing and hand wing is larger, a larger thrust is generated. This can also explain why a dragonfly will flap the fore wing and hind wing synchronously at the beginning of take-off, and the phase difference increases when the dragonfly is going to fly upward and forward.

It should be noted that current computational simulations are two-dimensional, whereas three-dimensional aerodynamic effects exist for the flapping of wings. However, this limitation does not affect the results and conclusions drawn above. Comparing the three-dimensional results (Sun and Lan^19^) with the two-dimensional results (Wang^24^; Lan and Sun^25^) of a hovering dragonfly (a situation similar to the current simulations), it can be shown that computed aerodynamic forces show relatively small differences under different dimensionalities. The averaged vertical force coefficient in three-dimensional simulations is approximately 20% less than that in two-dimensional simulations. This discrepancy is consistent with the lift reduction due to the tip vortex, which only exists in three-dimensions. Meanwhile, the time courses of the force coefficients in these simulations are nearly identical. This further indicates that regardless of whether the leading-edge vortex sheds or not, the difference in the vortex structures between two-dimensional simulations and three-dimensional simulations has little influence on the conclusions of this study.

In our simulations, we first focus on the variation in the angle of attack. The alternation of the pitching movement of the wing can be well represented in two dimensions, and the flow phenomenon induced by this alternation is primarily two dimensional. As for the phase difference, Russel and Wang^26^ studied its aerodynamic influence on a hovering dragonfly based on two-dimensional flow. The present research employs a similar approach and draws conclusions that are consistent with other two/three-dimensional simulations^19,25–27^. Three-dimensional effects definitely exist in the take-off flight of the dragonfly and will be of interest for future research.

## Conclusion

A dragonfly is airborne in approximately one wingbeat and then starts to take-off. The maximum vertical acceleration is close to 20 m/s^2^. The experimental result shows that the acceleration is generated only by aerodynamic force. The phase difference increases from approximately 0 degrees to 110 degrees. The angles of attack in the down-stroke of both wings in the first two wingbeats are significantly greater than those in the later wingbeats, resulting in different levels of aerodynamic lift.

The results of numerical simulation with the 2-D immersed boundary method for various angles of attack in the down-stroke and phase differences between the motion of the fore wing and hind wing show that flapping with a greater angle of attack in the down-stroke generates greater lift but smaller thrust; on the other hand, flapping with a lower angle of attack in the down-stroke induces greater thrust but smaller lift. Synchronous flapping (with zero phase difference) generates greater lift but smaller thrust. With the increase in the phase difference, larger thrust and smaller lift are induced.

Dragonflies are smart and good at using the best method of flapping. At the beginning of take-off, this requires rapidly increasing lift, so the flapping mode is synchronous with a large angle of attack in the down-stroke. After leaving the ground, smaller lift but larger drag is required to move forward, so the mode switches to flapping with increasing phase differences and decreasing angles of attack in the down-stroke.

## Methodology

### Experimental observation

#### Preparation of Animals

*Pantala flavescens* belongs to a widely distributed dragonfly family (Libellulidae) and is considered to be the most widespread dragonfly on the planet. Dragonflies were collected from a pond and lawn in Beihang University between 30 July and 16 August 2015. After this, the dragonflies were stored temporarily in a cool dark storage container and then moved into the indoor laboratory.

**Figure 12:**
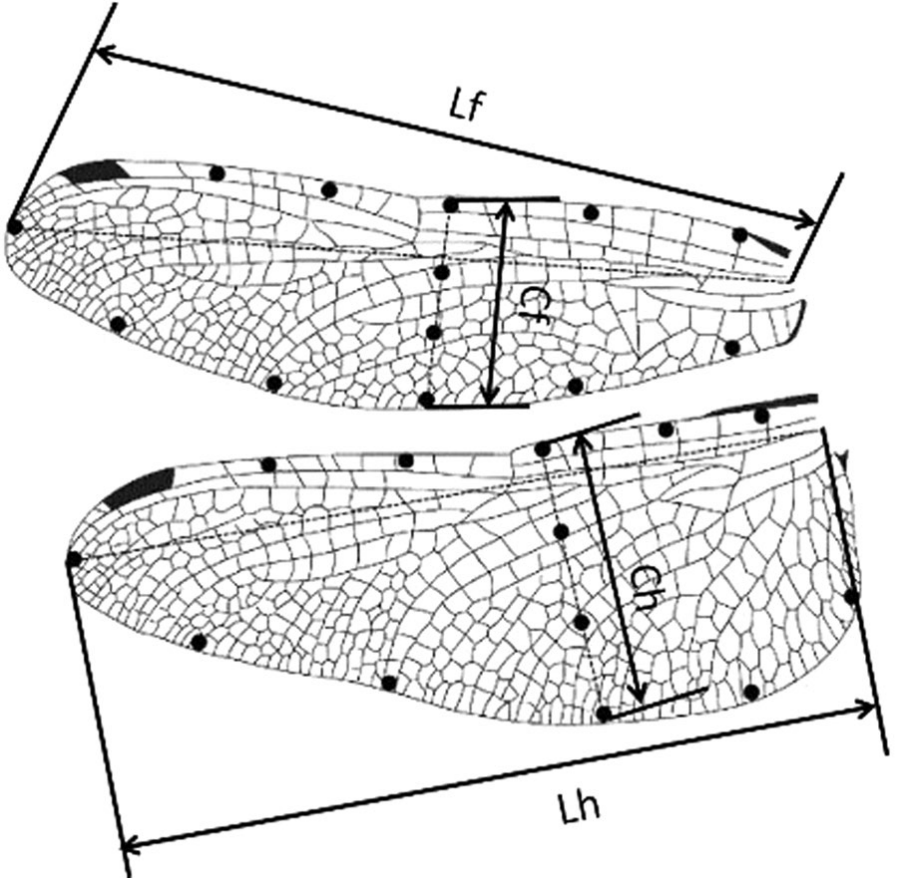
Wing mark point distribution; Cf, chord length of forewing; Lf, forewing length; Ch, chord length of hindwing; Lh, hindwing length.

In the laboratory, the dragonflies were first put in a refrigerator at a low temperature (~0–2 °C) for 1.5–2 hours. Then, the dragonflies were taken out and kept under anesthesia for approximately 5 minutes at room temperature. In this period, the featured parameters of dragonflies (such as weight and morphological parameters of the wings) were measured. For three-dimensional reconstruction, some black dots were marked on the wings (plotted in Fig. 12). All subjects were tested within 5 hours to ensure their activeness.

#### Experimental facilities

**Figure 13:**
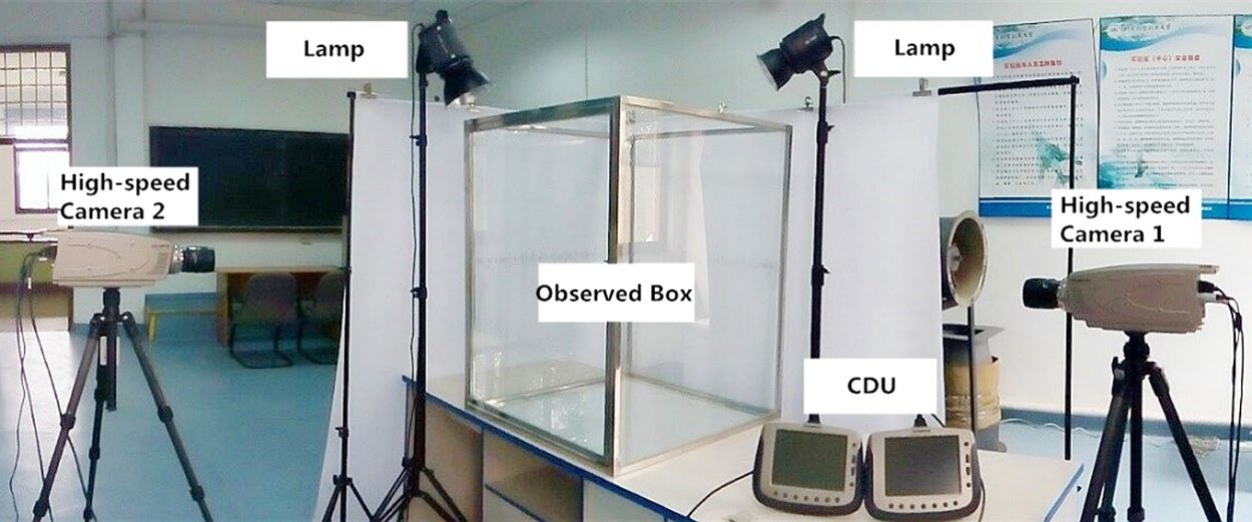
Experimental facilities.

As shown in Fig. 13, a 78 × 78 × 100 cm^3^ observation box made of iron frames and specular glass walls was used to limit the flying space of dragonflies. Two 1000 W photographic lamps were also mounted for illumination compensation in the filming area. Meanwhile, two high-speed cameras (Olympus i-SPEED TR, 1000 frames-1, shutter speed 1 ms, resolution 1280 × 1024 pixels) were used to film the take-offs of dragonflies.

#### Experimental procedure

Because the objective of this paper is to study the voluntary take-off of dragonflies, the subjects were individually put on the top of a support in the observation box. Then, videos were recorded as soon as each dragonfly moved its wings. For each dragonfly, the process of voluntary take-off was repeatedly captured 2–3 times. The total number of dragonflies tested in this experiment was 30. All the recorded voluntary take-off processes were almost the same, so the results shown in this paper of one dragonfly are representative.

#### Three-dimensional reconstruction

First, the optical axes of the cameras were set to be orthogonal to each other using a circular calibrator. Because the video recorded by each high-speed camera captures a two-dimensional plane, a three-dimensional reconstruction was performed to switch the image in the camera coordinates (*u*, *v*) to the world coordinates (*X*, *Y*, *Z*) using the linear coplanar method introduced in refs^28,29^.

The conversion formula is shown as follows:

1 $${Z}_{c}[\begin{array}{l}u\\ v\\ 1\end{array}]=[\begin{array}{ccc}1/dx & 0 & {u}_{0}\\ 0 & 1/dy & {v}_{0}\\ 0 & 0 & 1\end{array}]\,[\begin{array}{ccc}1+k{r}^{2} & 0 & 0\\ 0 & 1+k{r}^{2} & 0\\ 0 & 0 & 1\end{array}]\,[\begin{array}{cccc}\begin{array}{c}f\\ 0\\ 0\end{array} & \begin{array}{c}0\\ f\\ 0\end{array} & \begin{array}{c}0\\ 0\\ 1\end{array} & \begin{array}{c}0\\ 0\\ 0\end{array}\end{array}]\,[\begin{array}{cc}{\boldsymbol{R}} & {\boldsymbol{T}}\\ 0 & 0\end{array}]\,[\begin{array}{c}X\\ Y\\ Z\\ 1\end{array}]$$

where (*u*_0_, *v*_0_) are the coordinates of the main point of the camera, *dx* and *dy* are the size of pixels in the horizontal direction and vertical direction, respectively; *f* is the effective focal length; *k* is the distortion coefficient; *r* is the distance from the point to the optical axis; rotation matrix ***R*** and translation matrix ***T*** are calculated based on the calibration data; and *Z*_*c*_ is the field depth of the cameras.

**Figure 14:**
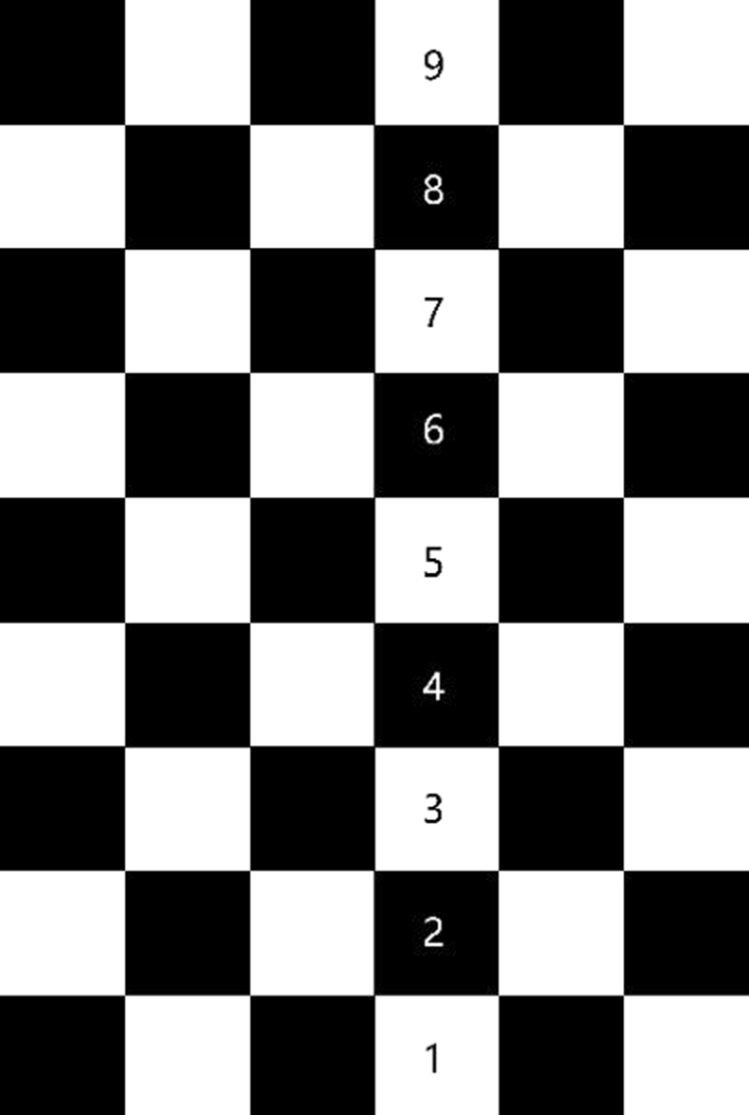
The board with known coordinate information used in the three-dimensional reconstruction, and the distance of adjacent points on this board is 100 mm.

A validation was carried out using a board with known coordinate information. The distance between two adjacent points on this board was 100 mm, as shown in Fig. 14. For the reconstruction, the maximum error was calculated to be 1.134 mm, corresponding to a maximum relative error of 1.13%. In the real test, the largest scale (the length of wings) was approximately 100 mm, so the maximum error was also estimated to be approximately 1.134 mm.

#### Kinematic parameter calculation

After the three-dimensional reconstruction, all coordinates of the dragonfly body and the marks on the wings can be obtained in the world coordinate system (*X*, *Y*, *Z*). However, the coordinates in a relative coordinate system (*X*_*b*_, *Y*_*b*_, *Z*_*b*_), located on the dragonfly, are also needed to obtain the kinematic parameters.

**Figure 15:**
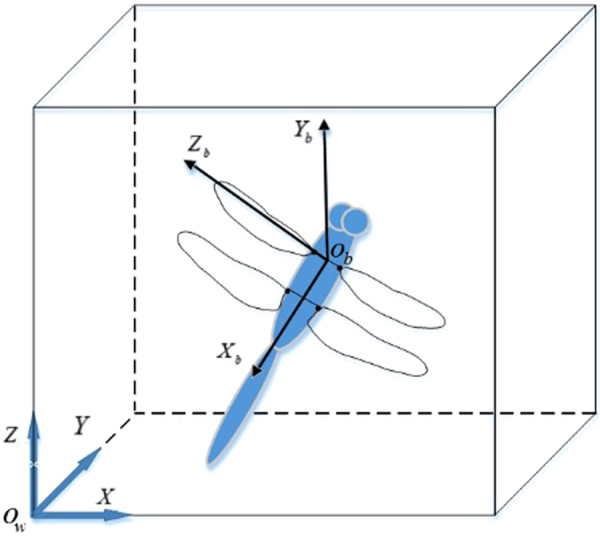
Transformation from the world coordinate system (*X, Y, Z*) to the relative coordinate system (*X*_*b*_*, Y*_*b*_*, Z*_*b*_).

Figure 15 shows the method we used to transform the world coordinate system to the relative coordinate system. The body centroid of the dragonfly is defined as the origin point (*O*_*b*_) of the relative coordinate system located on the dragonfly. Please note that the body centroid of the dragonfly is reasonably defined as the crossing point between the body axial line (from the head of the dragonfly to the tail) and the line connecting the root points of the right forewing and left forewing^18^. After that, the positive direction of the X-axis in the relative coordinate system is defined as the direction pointing toward the tail of the dragonfly and named the *X*_*b*_ direction. The Z-axis is set to be perpendicular to the *X*_*b*_ axis through the root points of both fore wings. Next, the Y-axis is readily defined because the relative coordinate system is a right-handed system.

To describe the wing kinematics of dragonflies, the method given by Ellington^30^ was followed. For the stroke plane of the fore wing, the coordinates (only *X*_*b*_ and *Y*_*b*_) of both fore wing tips in one wingbeat cycle were first marked on the plane of symmetry (plane *Z*_*b*_ = 0). Based on these projected points, a line can be determined by the linear regression method. As a result, the stroke plane of the fore wing is defined based on the wing tip line and the root of the wing. For the stroke plane of the hind wing, the same procedure was repeated.

**Figure 16:**
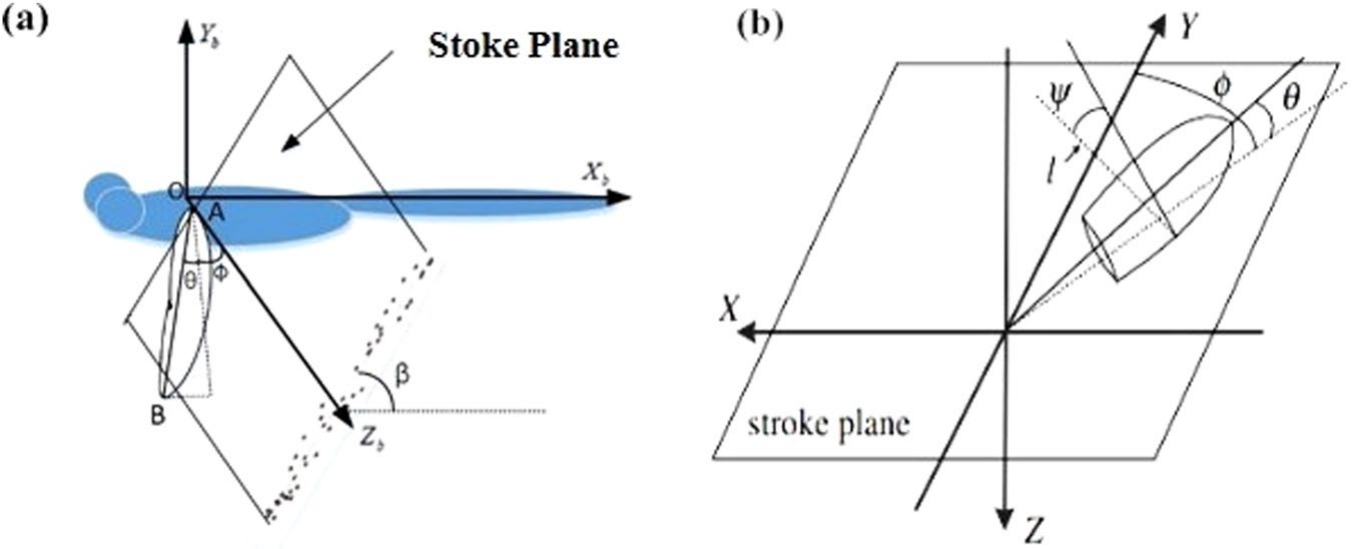
Definition of the angles of a flapping wing.

For each stroke plane, the angle between the stroke plane and the body axis is a constant, which is identified as the stroke plane angle β shown in Fig. 16a. In Fig. 16b, a line is drawn between the wing root and wing tip. Let (*X, Y, Z*) be a reference frame with the origin located at the wing root and the *X*–*Y* plane coinciding with the stroke plane. The orientation of the wing with respect to the stroke plane is determined by three Euler angles: the positional angle (*φ*), stroke deviation angle (*θ*) and pitch angle (*ψ*), where *φ* is defined as the angle between the Y-axis and the projection onto the stroke plane of the line joining the wing base and wing tip, *θ* is defined as the angle between the line joining the wing base and the wing tip and its projection onto the stroke plane, and *ψ* is defined as the angle between the local wing chord and line *l* (*l* is perpendicular to the wing span and parallel to the stroke plane). The angle of attack of the wing (*α*) has the following relationship with *ψ*: in the down-stroke, *α* = *ψ*; in the up-stroke, *α* = 180 degrees-*ψ*.

### CFD simulations

Because of the limitation of experimental approaches, CFD simulations of wing flapping were also carried out in this paper. The numerical method is introduced in detail as follows:

#### Immersed Boundary Method

The use of the orthogonal Cartesian coordinates is the most remarkable feature of the immersed boundary method (IBM), which makes the whole grid remain motionless regardless of the movement of the boundary. A source term is introduced into the governing equations to describe the effect of the boundary. The IBM was first applied to simulate cardiac blood flow in 1972^31^. Because of the great simplification of grid generation compared to traditional methods, especially in cases with transforming and moving boundaries, the IBM has been greatly developed and widely applied. Over the past 40 years, researchers have found a better way to address the boundary issues through upgrading and optimization in the IBM^32–34^.

The governing equations of the fluid domain are shown as follows:

2 $$\{\begin{array}{c}\frac{\partial {\bf{u}}}{\partial t}+({\bf{u}}\cdot \nabla ){\bf{u}}+\nabla p-\frac{1}{\mathrm{Re}}{\nabla }^{2}{\bf{u}}=0\\ \nabla \cdot {\bf{u}}=0\end{array}$$

where ***u*** is the fluid velocity, and *p* is the fluid pressure. The boundary velocity is given as a boundary condition: ***u*** = **μ**_Γ_. The boundary conditions cannot be applied directly to the boundary because the orthogonal Cartesian coordinates are employed in the IBM. Instead, the boundary conditions are incorporated into the governing equations by adding a source term. This paper chooses the IBM developed by Fladlun^35^ to simulate insect flapping flight. The method employs the discrete forcing approach to address the boundary, treats the interface between the solid and the fluid as a sharp interface, and obtains the grid coordinates intersecting with the boundary by determining the relative positions of the border and the grid.

#### Computational grid

**Figure 17:**
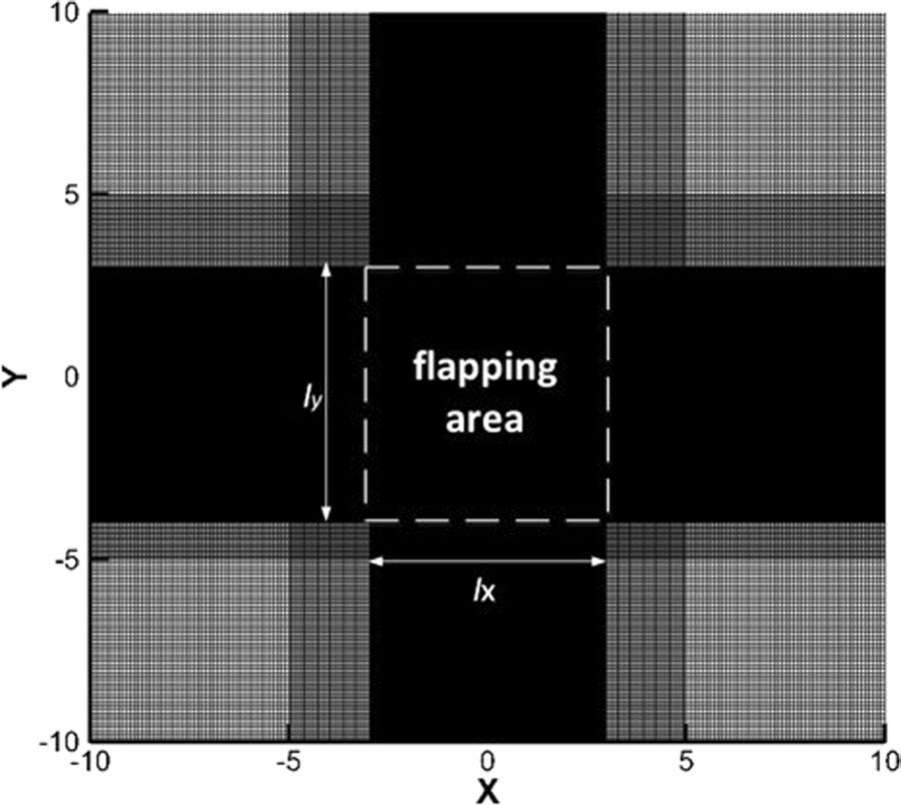
The computational grid.

The size of the entire computational domain is 20 × 20 (normalized by the chord of the wing *c*). A non-uniform grid is employed, and the domain is divided into three regions: the grid size of the outermost region is 0.1, that of the innermost region is 0.025, and that of the region in between is 0.05. The width and height of the innermost region are chosen in a manner that should encompass the flapping wing during the entire simulated process. The total grid number is 420 × 440. The computational grid is shown in Fig. 17. The time step is 1/800 (normalized by the cycle time). The Reynolds number (Re) is 157. The left side of the computational domain is the inflow boundary, and the other three sides are treated as outflow boundaries. At the inflow boundary, the velocity components are set equal to freestream values, and the Neumann pressure boundary condition is imposed. At the outflow boundary, Neumann boundary conditions are imposed for the velocities, while pressure is set equal to the freestream value. The ground effect is not considered in the simulations.

#### Rule of wing flapping

**Figure 18:**
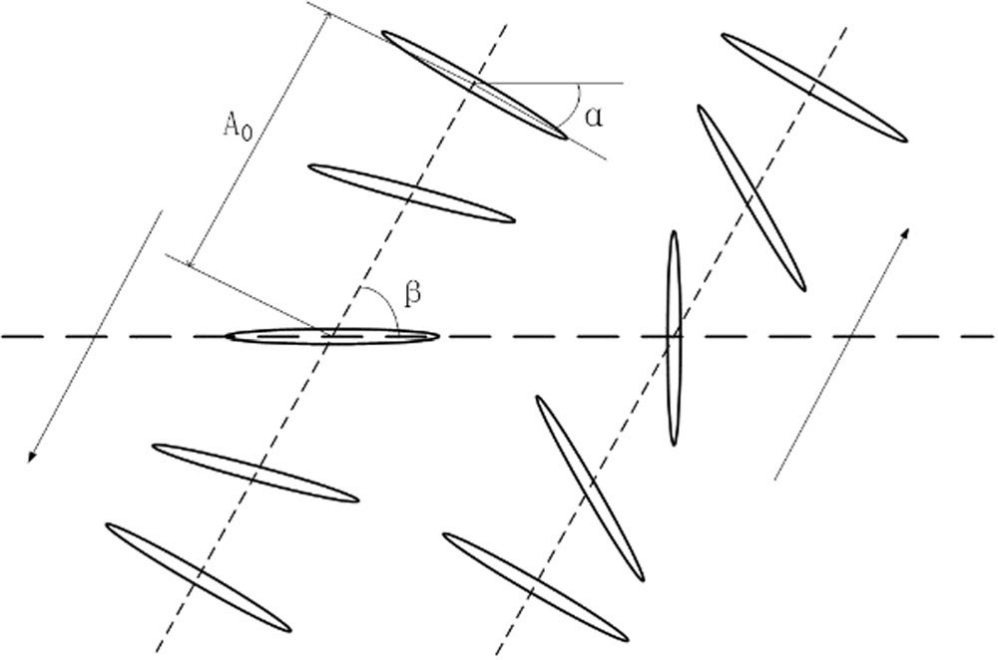
The model of flapping dragonfly wings.

In two dimensions, the flapping wing model used in this paper is as shown in Fig. 18. An ellipse whose ratio of the long axis to the short one is 10 is chosen to model the cross-section of a dragonfly wing in intermediate positions. The movement of the model wing can be simplified to two degrees of freedom: the translational movement of the center point of the model wing along the flapping plane and the rotational movement of the wing’s chord around the center of the chord^24^.

The translational movement can be expressed by the flapping amplitude defined as follows:

3 $$\{\begin{array}{c}{\Phi }_{f}=\frac{{A}_{f}}{2}\,\cos (2\pi ft)\\ {\Phi }_{h}=\frac{{A}_{h}}{2}\,\cos (2\pi ft+\phi )\end{array}$$

where *A*_*f*_ and *A*_*h*_ are the flapping amplitude of the fore wing and hind wing, respectively; *f* is the flapping frequency; and *φ* is the phase difference between the fore wing and the hind wing.

For the simulations, the coordinates of the center of the model wings can be written as follows:

4 $$\{\begin{array}{c}{x}_{f}(t)={x}_{fo}+\frac{{A}_{f}}{2}\,\cos (2\pi ft)\cos \,{\beta }_{f}\\ {y}_{f}(t)={y}_{fo}+\frac{{A}_{f}}{2}\,\cos (2\pi ft)\sin \,{\beta }_{f}\end{array}$$

5 $$\{\begin{array}{c}{x}_{h}(t)={x}_{ho}+\frac{{A}_{h}}{2}\,\cos (2\pi ft+\phi )\cos \,{\beta }_{h}\\ {y}_{h}(t)={y}_{ho}+\frac{{A}_{h}}{2}\,\cos (2\pi ft+\phi )\sin \,{\beta }_{h}\end{array}$$

where (*x*_*fo*_, *y*_*fo*_) and (*x*_*ho*_, *y*_*ho*_) are the initial positions of the fore wing and hind wing, respectively, and *β*_*f*_ and *β*_*h*_ are the stroke plane angles of the fore wing and hind wing, respectively.

According to the variation in the attack angle observed in real dragonflies in the experiment, the attack angle remains a stable value except at the beginning and the end of both up-strokes and down-strokes.

The rotational movement of the fore wing and the hind wing are described by Equation (6) and Equation (7), respectively, where α is the angle between the long axis of the ellipse and the horizontal plane, α_0_ is the initial rotation angle, α_m_ is the stable attack angle in the middle of flapping, and φ is the phase difference between the fore and the hind wing.

6 $$\begin{array}{c}\{\begin{array}{l}{\alpha }_{f}(t)={\alpha }_{0}+{\alpha }_{m}\,\sin (4\pi f)\\ 0 < t < \frac{T}{8}\,\frac{3T}{8} < t < \frac{5T}{8}\\ {\alpha }_{f}(t)={\alpha }_{m}\\ \frac{T}{8} < t < \frac{3T}{8}\,\frac{5T}{8} < t < \frac{7T}{8}\end{array}\end{array}$$

7 $$\begin{array}{c}\{\begin{array}{l}{\alpha }_{h}(t)={\alpha }_{0}+{\alpha }_{m}\,\sin (4\pi f+\phi )\\ 0 < t < \frac{T}{8}\,\frac{3T}{8} < t < \frac{5T}{8}\\ {\alpha }_{h}(t)={\alpha }_{m}\\ \frac{T}{8} < t < \frac{3T}{8}\,\frac{5T}{8} < t < \frac{7T}{8}.\end{array}\end{array}$$
